# Characterization of the virulence of a non-RT027, non-RT078 and binary toxin-positive *Clostridium difficile* strain associated with severe diarrhea

**DOI:** 10.1038/s41426-018-0211-1

**Published:** 2018-12-12

**Authors:** Chunhui Li, Céline Harmanus, Duolong Zhu, Xiujuan Meng, Shaohui Wang, Juping Duan, Sidi Liu, Chenchao Fu, Pengcheng Zhou, Ruisi Liu, Anhua Wu, Ed J. Kuijper, Wiep Klaas Smits, Lei Fu, Xingmin Sun

**Affiliations:** 1 Department of Infection Control Center of Xiangya Hospital, Central South University, Changsha, 410008 China; 2 Department of Molecular Medicine, Morsani College of Medicine, University of South Florida, 12901 Bruce B. Down Blvd, Tampa, FL 33612 USA; 3 Department of Medical Microbiology, Leiden University Medical Center, Leiden, The Netherlands; 4 Department of Infectious Disease of Xiangya Hospital, Central South University, Changsha, 410008 China

## Abstract

The expression of the *Clostridium difficile* binary toxin CDT is generally observed in the RT027 (ST1) and RT078 (ST11) *C. difficile* isolates, which are associated with severe *C. difficile* infection (CDI). However, we recently reported that the non-RT027 and non-RT078 *C.* difficile strain LC693 (TcdA^+^TcdB^+^ CDT^+^, ST201) caused severe diarrhea in a patient in Xiangya Hospital in China. *C.difficile* LC693 is a member of Clade 3, and in this study, we identified LC693 as RT871 and compared its virulence and pathogenicity to those of *C.difficile* R20291 (TcdA^+^TcdB^+^CDT^+^, ST1/RT027), UK6 (TcdA^+^TcdB^+^CDT^+^, ST35/RT027), CD630 (TcdA^+^TcdB^+^CDT^−^, ST54, RT012), and 1379 (TcdA^+^TcdB^+^CDT^−^, ST54/RT012), with strain 1379 being an epidemic *C.difficile* isolate from the same hospital. LC693 displayed a higher sporulation rate than R20291, CD630 or strain 1379. LC693 was comparable to R20291 with respect to spore germination, motility, and biofilm formation, but showed a faster germination rate, higher motility and a higher biofilm formation capability compared to CD630 and strain 1379. The adherence of spores to human gut epithelial cells was similar for all strains.The total toxin release of LC693 was lower than that of R20291, but higher than that of CD630 and strain 1379. Finally, in a mouse model of CDI, LC693 was capable of causing moderate to severe disease. Our findings demonstrate the pathogenicity of non-RT027 and non-RT078 binary toxin-positive *C. difficile* strains. Furthermore, our data indicate that LC693 may be more virulent than strain 1379, an epidemic strain from the same hospital, and provide the first phenotypic characterization of a non-RT027 and non-RT078 binary toxin-positive ST201 isolate.

## Introduction

*Clostridium difficile* (*C. difficile*) is the leading etiologic agent of antibiotic-associated diarrhea in developed countries and is emerging as a worldwide healthcare problem. Symptoms of *C. difficile* infection (CDI) range from mild diarrhea to severe pseudomembranous colitis, toxic megacolon, and death^1–3^. These symptoms are primarily caused by two large protein toxins, namely, TcdA and TcdB, which cause massive damage to the intestinal mucosa and induce acute inflammatory responses^4–6^. Approximately 20% of *C. difficile* strains also produce a third toxin,named the ADP-ribosyltransferase binary toxin (CDT)^7–9^. CDT increases the adherence of *C. difficile* to epithelial cells^10^ and suppresses colonic eosinophilia^11^. Although the exact role of CDT in the pathogenesis of CDI is not clear, hypervirulent *C. difficile* strains expressing CDT have been reported to cause a high mortality rate (approximately 60%)^8,9^.

In addition to toxins, factors such as spore formation, the presence of flagella and biofilm formation likely play important roles in *C. difficile* transmission and colonization^12–15^. CDI is transmitted via spores, which are metabolically dormant and highly resistant to a variety of disinfectants and antibiotics, allowing them to persist on surfaces outside of the host^12^. The persistence of spores is the root cause of the increased rates of recurrent CDI, and it is estimated that up to 33 and 45% of patients will develop relapse after the first and second episodes of CDI, respectively^16^. Although the role of flagella in *C. difficile* virulence may vary between strains, flagella play a role in adherence and colonization^17,18^. Bacteria in biofilms are known to be more resistant to different environmental stresses, including antibiotics^19^. Interestingly, *C. difficile* R20291, a PCR ribotype (RT)027 strain, produces higher levels of toxins in vitro than CD630^20^ and has increased sporulation and biofilm formation capabilities^15^.

*C. difficile* strains carrying the binary toxin (CDT) are generally found among lineages that include the epidemic RT027 (multilocussequence type (ST)1) and RT078 (ST11) strains, which are associated with severe clinical symptoms in North America and Europe^21^. However, both RT027 and RT078 strains are rarely reported in China, with RT027 strains not having been reported in China until 2013, and RT078 strains have not been reported to date^22^. Instead, several toxigenic *C. difficile* strains associated with severe symptoms have been reported that belong to different clades^23,24^, indicating that the epidemic genotypes of *C.difficile* in China are different from those in North America and Europe. Previously, we reported a severe CDI case caused by the binary toxin-positive, non-RT027, and non-RT078 *C. difficile* strain LC693 belonging to ST201^22,25^. In this study, several potential virulence factors of strain LC693 were evaluated in vitro and in vivo and were compared to those of RT027 (ST1) and RT012 (ST54) strains.

## Results

### Strain LC693 is PCR ribotype 871 (RT871)

In our previous study, we showed that strain LC293 belongs to Clade 3, ST201^22,26^. Multiple clade 3 strains have been described^23^, with the most common Clade 3 RT in Europe being RT023 (3%)^27,28^. Similar to LC693, RT023 strains are TcdA^+^, TcdB^+^, and CDT^+^ strains. We performed capillary PCR ribotyping^29^ on LC693 against the database of the Dutch National Reference Laboratory, a center recognized by the European Centre for Disease Prevention and Control (ECDC) for the typing of *C. difficile*. LC693 was determined to be an RT871 strain, whereas the epidemic 1379 strain was readily confirmed as an RT012 strain, consistent with its ST54 designation.

### Strain LC693 efficiently sporulates

**Fig. 1:**
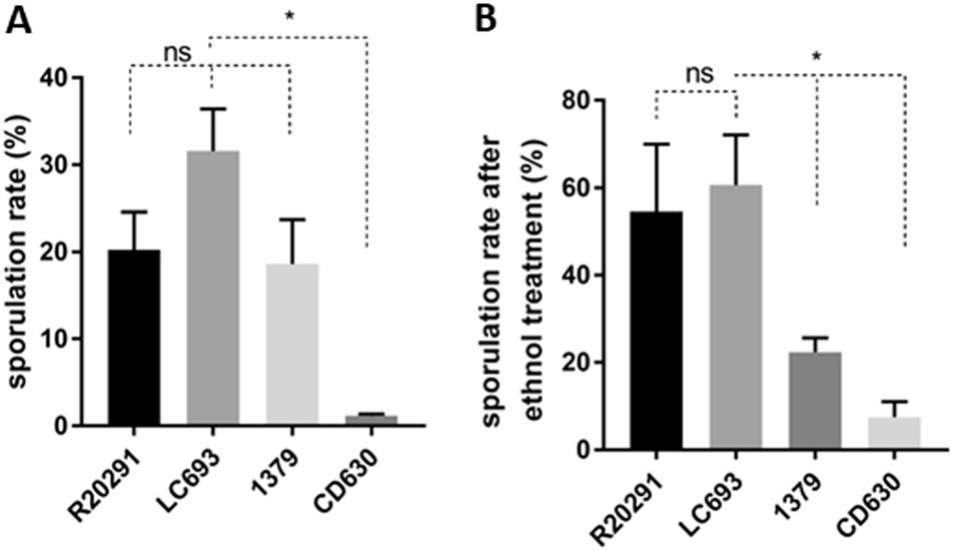
Strain LC693 efficiently sporulates. *C*. *difficile* strains were grown in 70:30 liquid sporulation medium and assayed for spore formation after 24 h of incubation. **a** Sporulation frequencies were plotted from direct enumeration of spores and vegetative cells in micrographs (data not shown). **b** Sporulation frequency determined by ethanol inactivation of vegetative cells. The means ± standard deviation of the mean from three biological replicates are shown. Student’s t-test was used to assess significance; ns not significant; *p < 0.05

To evaluate the sporulation rate of *C. difficile* strains, bacteria were cultured in 70:30 sporulation medium and assessed for sporulation rates at 24 h post inoculation (T_24_). For comparison, we evaluated the sporulation of the reference strains R20291 (TcdA^+^TcdB^+^ CDT^−^; RT027/ST1)^30^, CD630 (TcdA^+^TcdB^+^ CDT^−^; RT012/ST54), and 1379 (TcdA^+^TcdB^+^ CDT^−^; RT012/ST54), the latter of which is an epidemic strain isolated from the same hospital as LC693. R20291 was included as a well-characterized control for a binary toxin-positive epidemic strain. An RT078 strain was not included, as phylogenetic analyses demonstrated that clade 3 strains are more closely related to clade 2 (RT027/ST1) than clade 5 (RT078/ST11)^31,32^. Samples were visualized by microscopy (data not shown), and the sporulation frequency was defined as the number of spores divided by the number of vegetative cells. As shown in Fig. 1, strain LC693 exhibited a high sporulation rate (31.59 ± 4.82%), significantly exceeding that of CD630 1.17 ± 0.24% (*p* = 0.0124). LC693 also showed a higher sporulation frequency than R20291 and strain 1379, although these differences were not significant (LC693, 31.59 ± 4.82% versus R20291, 20.27 ± 4.34%, *p* = 0.1322; and versus strain 1379, 18.61 ± 5.11%, *p* = 0.1205).

Similar results were obtained when the sporulation of strains was examined by the frequency of ethanol-resistant spore formation rather than by microscopic enumeration. LC693 exhibited a high frequency of ethanol-resistant sporulation 60.63 ± 11.49%, similar to that observed for R20291 (54.55 ± 15.43%, *p* = 0.6986). Strains CD630 and 1379 displayed a much lower level of ethanol-resistant sporulation, 7.50 ± 3.54% and 22.34 ± 2.34%, respectively (LC693 vs CD630, *p* = 0.0247; LC693 vs 1379, *p* = 0.0454).

Thus, LC693 sporulation is on par with the RT027 strain and is significantly higher than that of the RT012 strains under the conditions tested.

### LC693 spores readily germinate

**Fig. 2:**
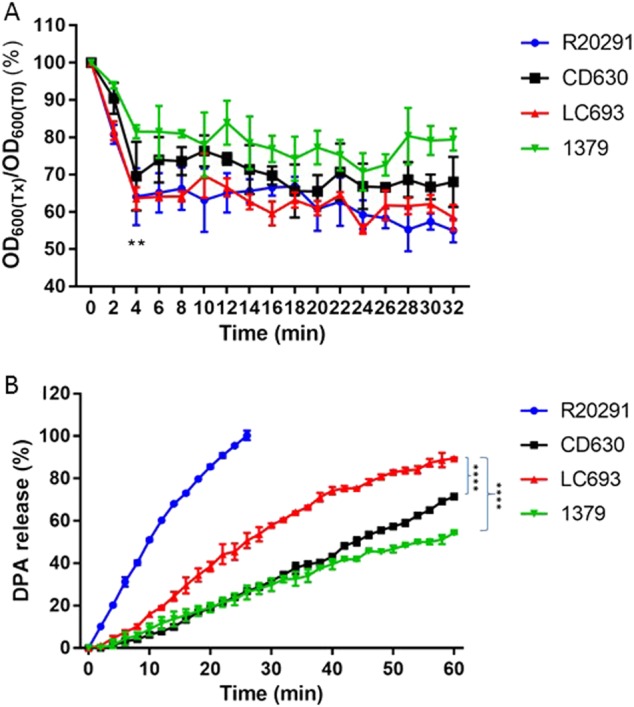
LC693 spores readily germinate. Purified *C. difficile* spores were suspended in buffer supplemented with taurocholic acid and glycine to induce germination. **a** Germination was monitored by plotting the ratio of the OD_600_ at a given time to the OD_600_ at time zero. **p = 0.0001, LC693 vs CD630 and strain 1379, one-way ANOVA. **b** CaDPA release from germinating *C. difficile* spores was monitored using Tb^3+^-fluorescence, and values were normalized to the maximum signal obtained for strain R20291 at 30 min. (****p < 0.0001). Data are reported as the means ± SD from three independent experiments

To compare early events in spore germination, we incubated purified *C. difficile* spores in a buffer supplemented with glycine and taurocholic acid^33^. Germination was monitored by plotting the ratio of the optical density at 600 nm (OD_600_) at a given time to the OD_600_ at T_0_ (time zero). We observed a rapid decrease in optical density indicative of germinating spores within the first 4–6 min (Fig. 2a). This decrease was similar at 4 min for LC693 (63.57 ± 3.01%) and R20291 (64.08 ± 7.64%, *p* = 0.9195), but significantly greater than that observed for CD630 and strain 1379 (69.55 ± 9.21% and 81.48 ± 1.79%, respectively, *p* = 0.0001, using one-way ANOVA).

Germination is accompanied by the release of calcium dipicolinic acid (CaDPA) from spores^18,19^. A previous study showed the complete release of CaDPA from *C. difficile* spores in the presence of taurocholic acid and glycine within ~30 min^34^. We monitored CaDPA release in real-time using an assay based on terbium fluorescence^35^ (Fig. 2b). Consistent with previous studies, we observed that R20291 exhibited a 100% CaDPA release after approximately 30 min. The fluorescence signals from the other strains did not saturate within this time frame and were monitored for 60 min, at which time LC693 reached an 89.48 ± 0.41% CaDPA release, which was significantly higher than that observed for strains CD630 and 1379 (*p* < 0.001) (Fig. 2b).

Taken together, these results indicate that LC693 spores more readily germinate than those of strains 630 and 1379.

### LC693 is motile with abundant peritrichous flagella

**Fig. 3:**
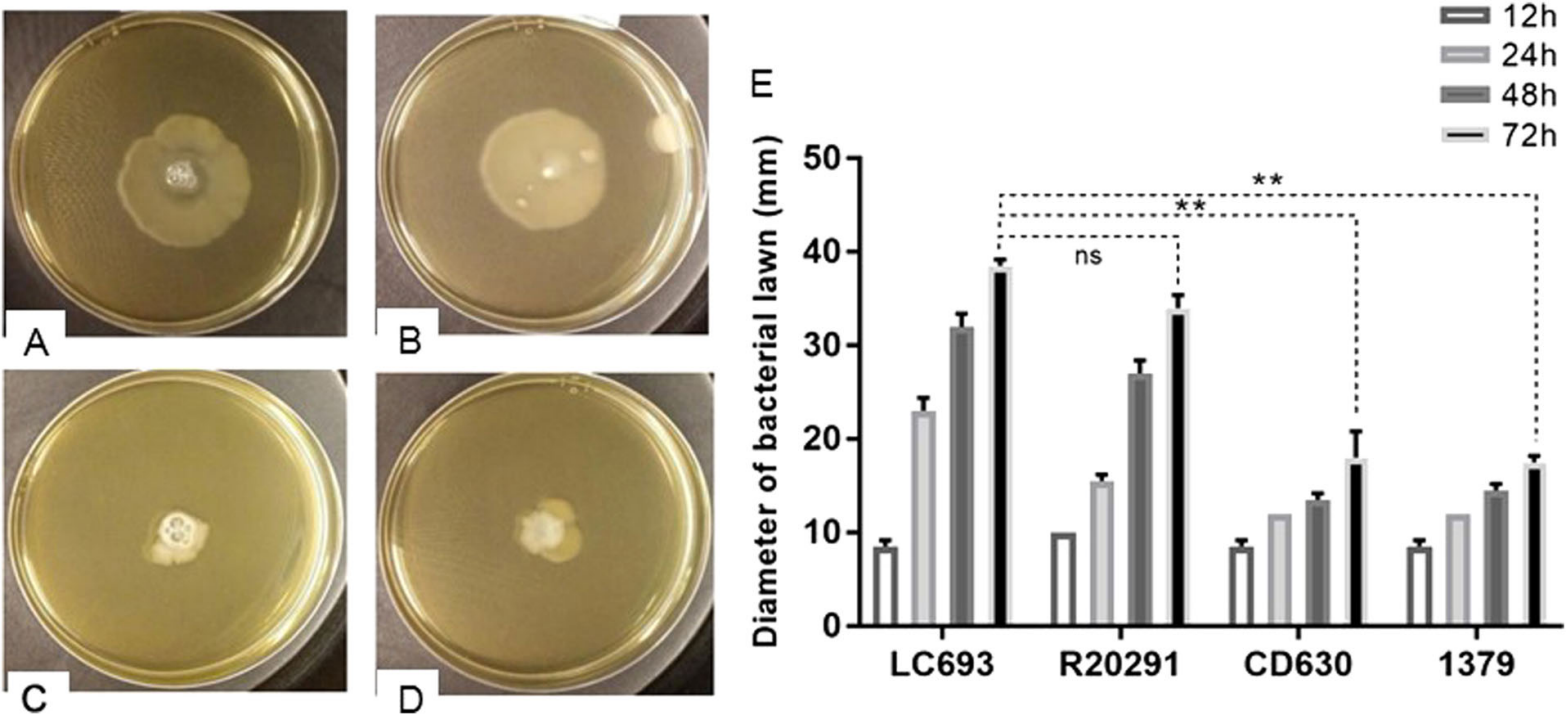
LC693 is motile. A single colony was inoculated in the middle of a swim plate, which was incubated at 37 °C for the indicated time. Swimming motility was quantitatively determined by measuring the radius of the zone of motility derived from a single colony. **a**–**d** Bacterial growth resulting from strains LC693 (**a**), R20291 (**b**), CD630 (**c**) and 1379 (**d**) after 72 h of incubation (**e**). Radius of the bacterial growth derived from a single colony of each strain. Data are reported as the means ± SD from three independent experiments, and differences were analyzed by Student’s *t*-test; ns not significant; **p < 0.001

To assess the motility characteristics of *C. difficile* vegetative cells, swimming assays were performed on 0.3% nutrient agar plates. The radius of the bacterial growth after incubation is correlated to swimming motility. Strains LC693 (38.5 ± 0.71 mm) and R20291 (34 ± 1.41 mm) produced similar radii (Fig. 3) (*p* = 0.07), whereas strains 1379 and CD630 appeared to be significantly less motile, producing radii of 17.5 ± 0.71 and 18 ± 2.83 mm, respectively LC693 vs CD630, *p* *=* 0.0036; LC693 vs 1379, *p* = 0.0041).

**Fig. 4:**
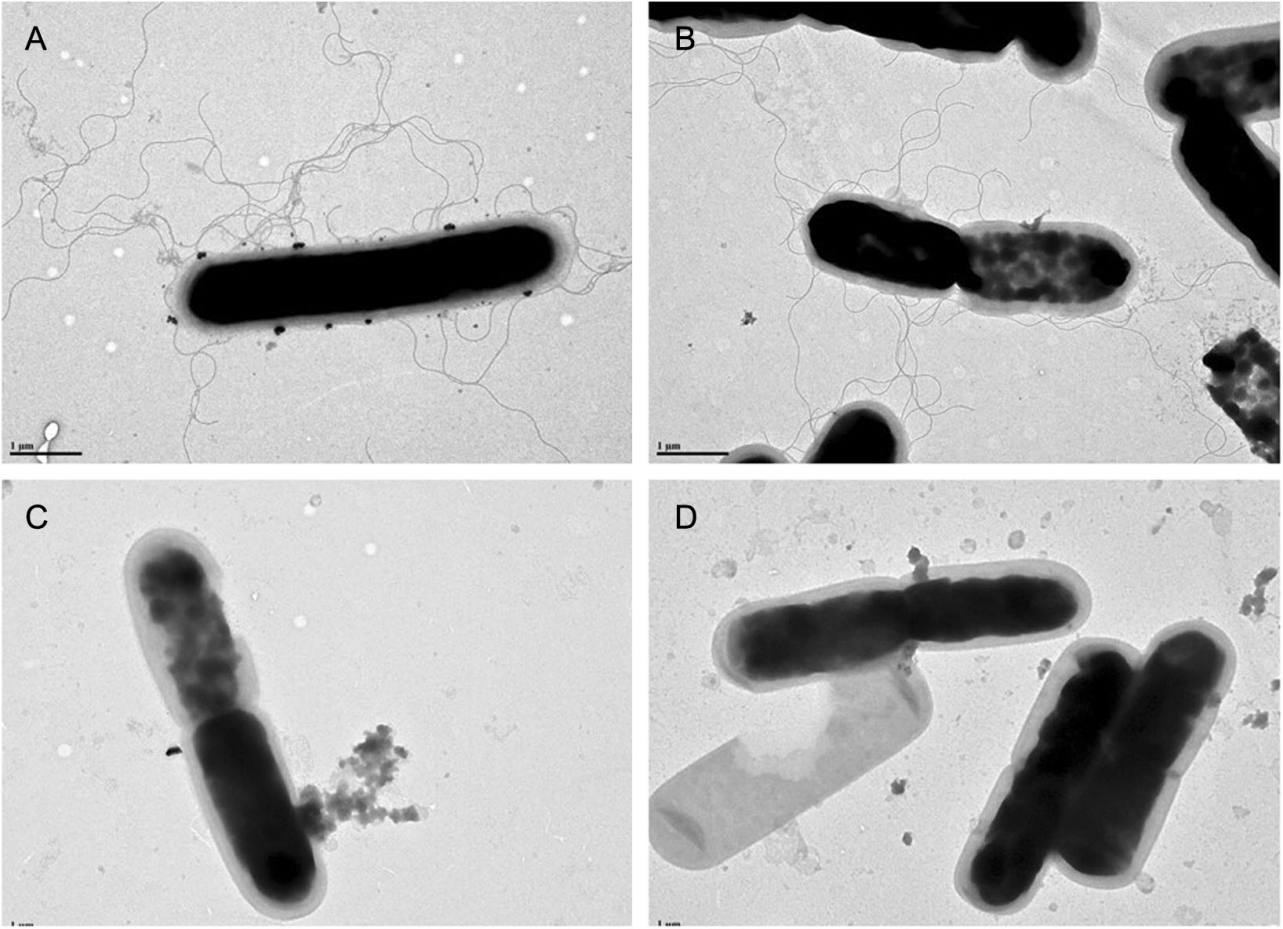
LC693 forms peritrichous flagella. Flagella were visualized by transmission electron microscopy. Scale bar represents 1 μM. **a** LC693; **b** R20291; **c** CD630; and **d** 1379

We related the motility phenotype to the presence of flagella through a transmission electron microscopy (TEM) analysis. Consistent with our motility assay, we observed that LC693 and R20291 cells presented abundant peritrichous flagella, whereas CD630 and 1379 cells did not appear to produce flagella (Fig. 4).The presence of flagella therefore appears to correlate with the strong swimming motility observed for strains LC693 and R20291.

### LC693 is highly capable of biofilm formation

**Fig. 5:**
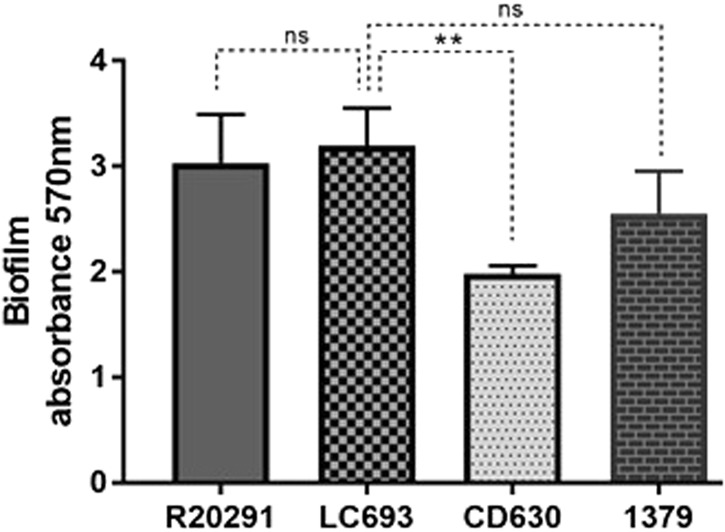
LC693 can form a biofilm. Biofilm formation was measured by crystal violet staining after 5 days of growth in reinforced clostridial medium. Data are from three biological replicates and are reported as the means ± SD. Student’s *t*-test was used for assess significance; ns not significant; **p < 0.001

The capability of the different *C. difficile* strains to form biofilms was evaluated by crystal violet (CV) staining after growth in microtiter plates^36^. Our results showed that after incubating for 5 days, strain CD630 formed the least amount biofilm on the abiotic surface, while all the other assayed strains, including R0291 and LC693, exhibited comparable levels of biofilm formation (Fig. 5). These results indicate that LC693 is a competent biofilm-forming strain.

### LC693 spores adhere well to human gut epithelial cells

**Fig. 6:**
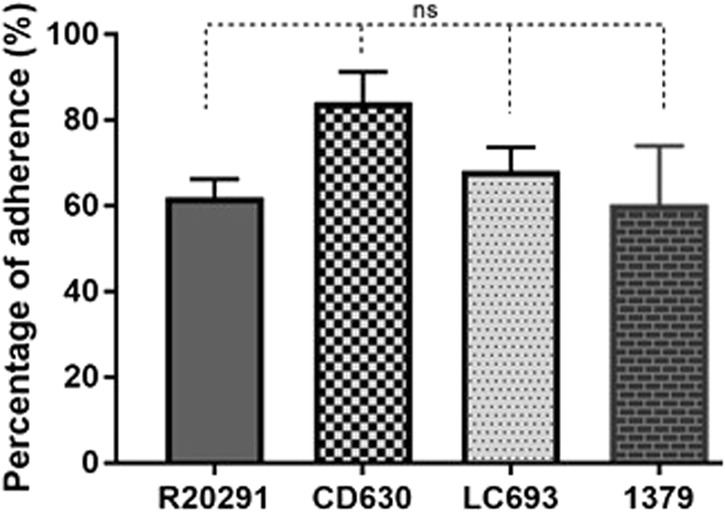
LC693 spores adhere well to human gut epithelial cells. HCT-8 cells were incubated with spores for 100 min under anaerobic conditions, and the relative amount of adhered spores was subsequently calculated. ns: not significant

Purified *C. difficile* spores were used to evaluate their adherence to HCT-8 human gut epithelial cells. We observed no significant differences in spore adherence among the strains (Fig. 6) (one way ANOVA: *p* = 0.136). CD630 showed the highest adherence rate (84.19% of adherent spores), whereas that observed for LC693, R20291 and strain 1379 averaged approximately 60%.

### Strain LC693 is an effective toxin producer

The primary virulence factors of *C. difficile* are TcdA and TcdB, which are encoded in a pathogenicity locus together with the genes *tcdR*, *tcdE,* and *tcdC*^37^. *C. difficile* clade 3 strains, including LC693, contain the mobile genetic element Tn*6218*, which is inserted between the *tcdE* and *tcdA* genes^23^. In this assay, we used both a conventional ELISA and a commercial toxin detection kit to assess extracellular TcdA/TcdB release to determine whether this insertion leads to aberrant toxin expression in strain LC693.

**Fig. 7:**
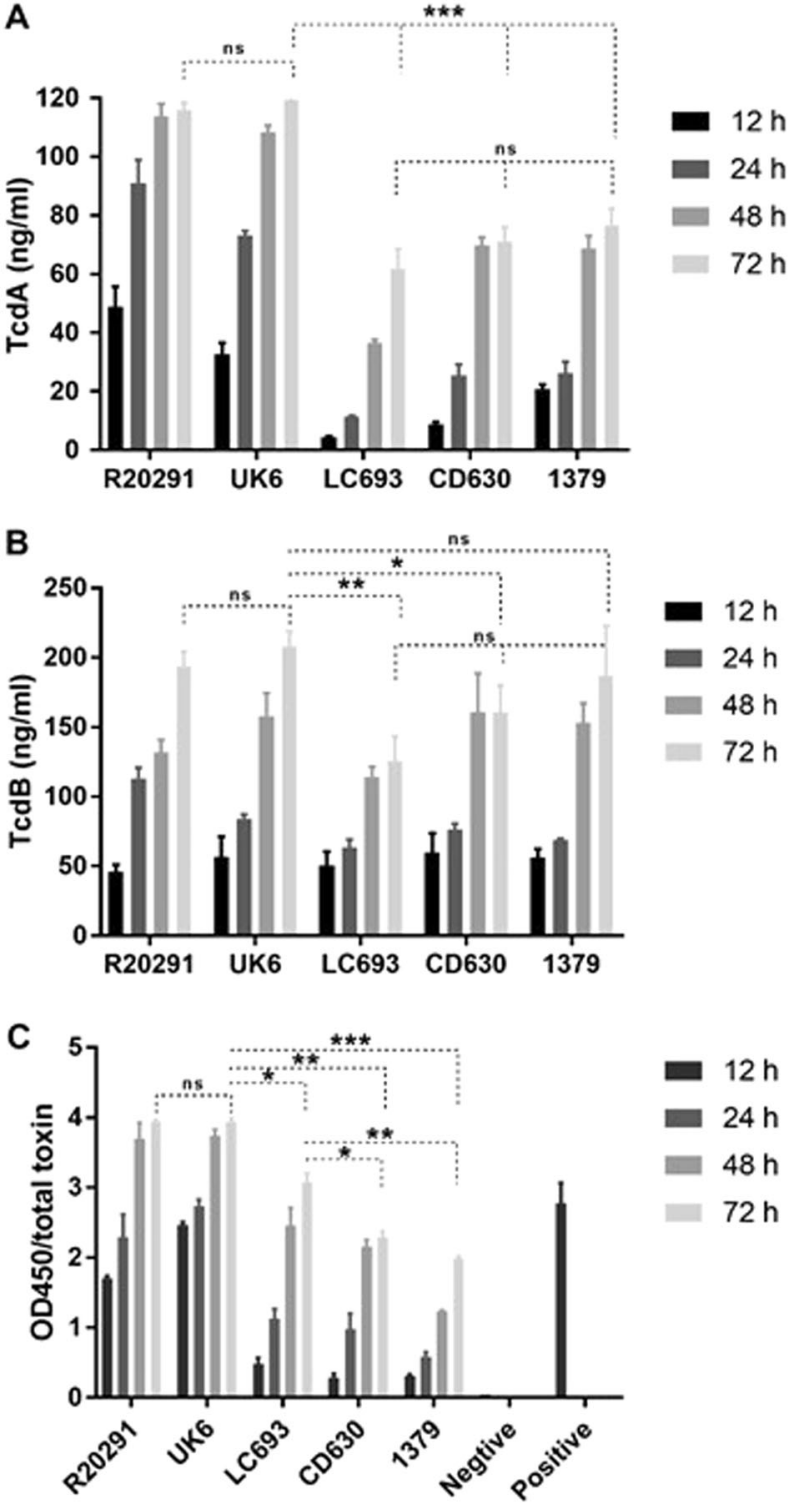
LC693 releases TcdA and TcdB. Toxin production at four different time-points was measured. **a** TcdA concentration in culture supernatant as determined by conventional ELISA; **b** TcdB concentration in culture supernatant as determined by conventional ELISA; **c** Total toxin released into the supernatant as measured using the *C. DIFFICILE TOX A/B II*™ assay. Student’s *t*-test was used for to assess significance; ns not significant; *p < 0.05; **p < 0.001; ***p = 0.0001

Strain UK6 (A^+^B^+^CDT^+^) is a hypervirulent BI/027/NAP1 *C. difficile* strain that is routinely used for a mouse model of CDI^38,39^. Since we used strain UK6 in the CDI model as a reference strain, UK6 was also included in the toxin assays. In the conventional ELISA assay, all the assayed strains produced both TcdA and TcdB, with the highest toxin concentrations of TcdA and TcdB detected at 72 hours postinoculation (Fig. 7a, b). Generally, TcdA levels (Fig. 7a) were lower compared to TcdB levels (Fig. 7b). Importantly, the dynamics of toxin release into the medium were similar between LC693 and the other strains overall, suggesting that the insertion of Tn*6218* does not markedly influence toxin regulation or release (Fig. 7). At 72 h postinoculation, the TcdA and TcdB levels did not significantly differ between the RT027 strains (R20291 and UK6). For TcdA, we observed that the RT027 strains produced approximately 2-fold more toxin than LC693 and the RT012 strains (CD630 and 1379) at 72 h postinoculation. For TcdB, toxin levels were similar for the RT027 strains and strain 1379, but lower for LC693 (*p* < 0.0001) and CD630 (*p* = 0.0109) at 72 h postinoculation.

Total toxin (i.e., the combined amount of measured TcdA and TcdB released into the medium) determined using the *C. DIFFICILE* TOX A/B II™ assay confirmed that high levels of toxins were produced by the RT027 strains (R20291 and UK6) under the conditions tested (Fig. 7c). Although LC693 showed a lower toxin production compared with R20291 and UK6 (*p* < 0.05), it produced significantly higher amounts of total toxin than strains CD630 and 1379 (*p* = 0.0181 and *p* = 0.0091, respectively) (Fig. 7c). The observed difference using the conventional ELISA may be related to the sensitivity of the antibodies used in the assay.

Taken together, our data indicate that the temporal regulation of toxin release by strain LC693 mirrors that of other *C. difficile* strains that lack the Tn6218 insertion. Furthermore, this strain produces toxins at a level that is similar to or exceeds that of the RT012 strains tested.

### Evaluation of LC693 virulence in a mouse model of CDI

**Fig. 8:**
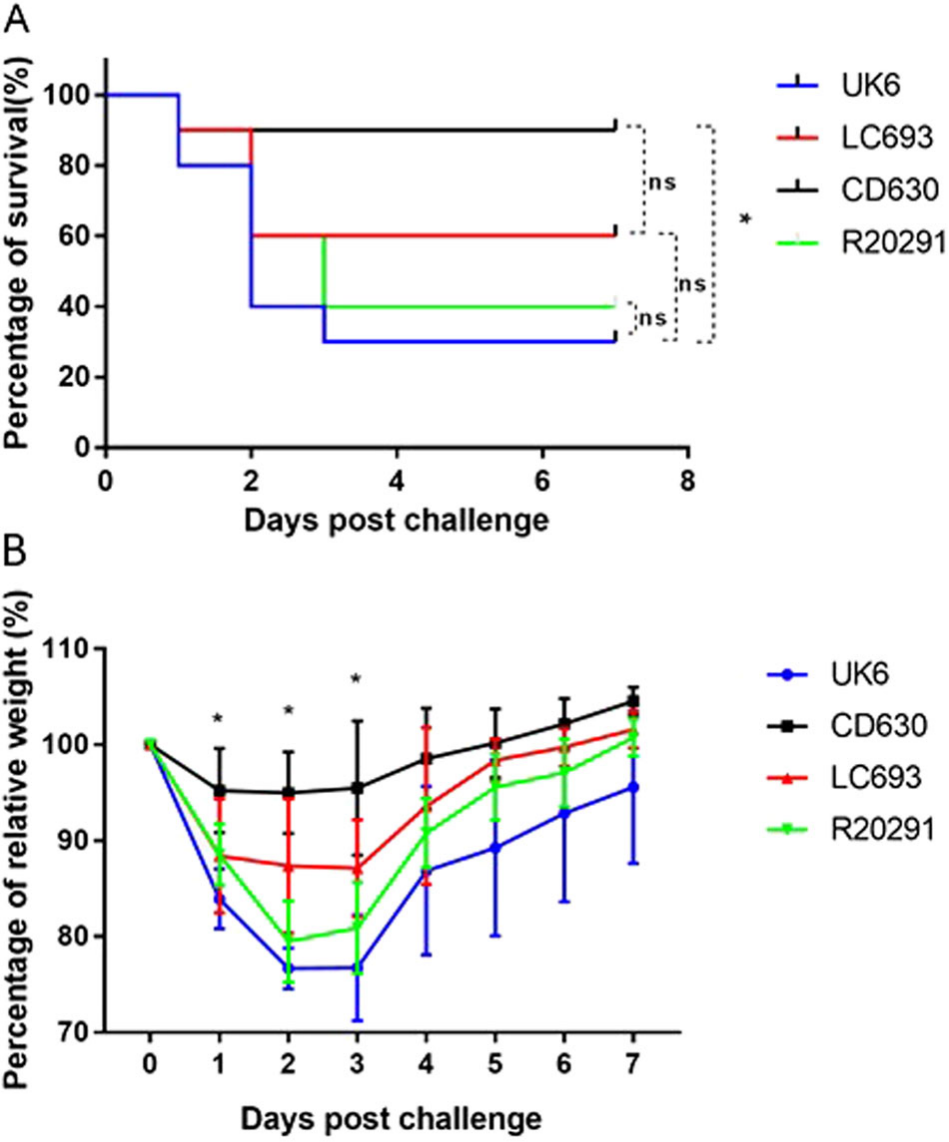
LC693 is virulent in a mouse model of CDI. Four groups of mice (*n* = 10) were challenged with 10^6^ spores of UK6, LC693, CD630, or R20291. Mice were monitored for one week for survival (**a**) and weight changes (**b**). Animal survival was analyzed by Kaplan–Meier survival analysis with a log-rank test of significance (ns not significant, *p* *>* 0.05). The mean relative weight of mice was analyzed for significance using a Student’s *t*-test. *p < 0.05 between the CD630 and LC693 groups at 1, 2 and 3 days post challenge; *p* < 0.05, between the CD693 and UK6 groups at 2, 3, 5, 6, and 7 days post challenge; *p* < 0.05 between the LC693 and R20291 groups at 2 and 3 days post challenge; and *p* > 0.05 between the UK6 and R20191 groups at all assayed time points

Ultimately, the traits investigated above are likely to play a role in disease development and pathogenesis. Therefore, we evaluated the pathogenicity of LC693 in mouse model of CDI. We did not include strain 1379 in the animal experiments, since we sought to focus on comparing the virulence between LC693 and the hypervirulent RT027 strains and the *C. difficile* strain CD630. In a separate report, we will publish a more detailed study of strain 1379, which was beyond the scope of the current report. We observed that only a single animal succumbed to a challenge with CD630, whereas mortality was higher (40–70%) with the other assayed strains (Fig. 8). Although the LC693-challenged group showed a trend toward higher mortality (40%), this intermediate level was not significantly different from the CD630- or RT027 (R20291 and UK6)-challenged groups. Notably, the RT027-challenged group showed significant higher mortality compared to the CD630-challenged group (*p* < 0.05).

When monitoring weight loss as an indicator of disease severity, we observed that CD630-challenged mice showed only moderate signs of disease, in line with the low mortality observed for this group (Fig. 8b). In contrast, the groups challenged with the other strains showed a sharp decrease in body weight at 1–3 days post challenge. Weight loss in the LC693-challenged group at 1, 2, and 3 days post infection was significantly greater than was observed for the CD630-challenged group. The LC693-challenged group lost significantly more weight at 2, 3, 5, 6, and 7 days post infection and at 2 and 3 days post infection compared to UK6- and R20291-challenged groups, respectively (*p* < 0.05) (Fig. 8b). Overall, the results from the mouse model of CDI suggest that LC693 is capable of causing moderate to severe disease.

## Discussion

RT027/NAP1/ST1 *C. difficile* isolates have rapidly emerged in the past decade as epidemic strains in North America and Europe, resulting in CDI with high morbidity and mortality in hospitalized patients. In addition, the emerging RT078/ST11 *C. difficile* strains have been reported to cause severe CDI at a similar rate^40^. Epidemic RT027 strains have been associated with more severe disease because of their increased production of TcdA and TcdB (due to a *tcdC* deletion)^40,41^, high-level fluoroquinolone resistance and efficient sporulation^42^. However, in China, RT027 strains have not been reported until 2013, and RT078 strains have not been reported to date^22^. Instead, several severe symptoms associated with toxigenic *C. difficile* strains belonging to clades other than RT027 and RT078 have been reported^23,24^, indicating that the epidemic genotypes of *C. difficile* in China are different from those in North America and Europe.

LC693 belongs to clade 3 and ST201. A previous phylogenetic analysis showed that clade 3 strains have a closer evolutionary relationship with the RT027 strains than with RT078 strains^25^, which is why we included RT027 strains in our comparative characterization of LC693.We showed that LC693 is an RT871 *C. difficile* strain that sporulates well, germinates rapidly, readily forms biofilms, and forms spores that are capable of adhering to human gut epithelial cells. Importantly, LC693 caused severe symptoms in both mice, as shown in this work, and humans (from which it was previously isolated).

To be able to produce toxins and cause disease symptoms, *C. difficile* spores need to germinate into vegetative cells, making germination an important virulence feature. Spore germination efficiency for severe CDI-implicated *C. difficile* strains was shown to be significantly higher than for strains associated with nonsevere CDI^43^. In addition, *C. difficile* spores with a high germination rate were observed to be more likely to cause recurrent CDI^44^. We showed that strain LC693 efficiently sporulates and germinates (Figs. 1 and 2), indicating its high potential in promoting CDI.

Flagella are important colonization factors for a number of enteropathogenic species, including *C. difficile*^15^. Flagella may also play an important role in allowing bacteria to adapt to their unique biological niches^13,45^. The results of our study demonstrated that strain LC693 is highly motile (Fig. 3), which was supported by the observation that it had abundant peritrichous flagella (Fig. 4), similar to the well-characterized epidemic RT027 isolate R20291. Strikingly, these strains were also highly similar in their capacity to form biofilms (Fig. 5), which may contribute to their in vivo virulence^45^. We noted that our results for strains CD630 and R20191 appear to be different from those observed by others with respect to motility and flagella production^47^. Baban and coworkers^47^ reported that CD630Δerm, an erythromycin sensitive strain derived from CD630, was peritrichously flagellated and that R20291 was monotrichously flagellated with only a single flagellum. This group also showed the R20291 displayed decreased motility compared to 630Δerm in swimming and swarming assays. Although we do not know the reason for this discrepancy, differences in experimental methods, minor differences in genetic background^47^, and possible phase variation of flagellar gene expression^48,49^ could contribute to the differences in results. Our swimming motility results were consistent with the TEM images. In addition, work from our laboratory and others has shown that even seemingly identical strains may not be genetically identical^50^. Whereas we used strain CD630, the authors in the other study used 630Δerm^47^, a strain derived from strain CD630 by serial passaging. Finally, even an isogenic strain can behave differently due to reasons such as phase variation^48,49^. In agreement with our results, Stabler et al.^30^ showed that CD630 was less motile compared to R20291, and Anjunwon-Foster and Tamayo^48^ showed that a “flagellar switch” controlled the phase variable production of flagella and toxins in R20291, which displayed peritrichous flagella with the flagellar switch on.

TcdA and TcdB are the major cause of CDI symptoms^6,51^, and the greater severity of RT027-associated CDI has been partially attributed to the production of higher amounts of toxins, which may or may not be caused by mutations in *tcdC*^50,52^. The results of our previous study demonstrated that the toxin-encoding genes of strain LC693 were highly homologous to those of epidemic RT027/ST1 strains^25^. Although the release of toxin by LC693 did not reach the levels observed for the RT027 strains included in this work (UK6 and R20291), the mortality for the LC693-challenged group (40%) was not significantly different from that of the RT027 strains. Furthermore, low toxin-producing *C. difficile* strains with higher colonization capability are able to cause severe infection outcomes (meeting presentation by Dr. Gayatri Vedantam). In agreement with the observed virulence of LC693 in the mouse model of CDI, our previous case report also showed that this strain caused severe diarrhea in an ICU-hospitalized patient^22^.

In summary, our data highlight the potential risk that ST201 strains can cause epidemics with severe outcomes in China.

## Materials and methods

### Measurement of sporulation rate

The microscopic evaluation of sporulation was performed as previously described^33^. *C. difficile* strains were cultured to mid-log phase in BHIS medium supplemented with 0.1% taurocholate (Sigma) and 0.5% fructose at 37 °C in an anaerobic chamber. A mixture of 70:30 sporulation medium (70% SMC medium and 30% BHIS medium containing 63 g Bacto peptone, 3.5 g protease peptone, 11.1 g BHI medium, 1.5 g yeast extract, 1.06 g Tris base, 0.7 g NH_4_SO_4_, and 15 g agar per liter) was prepared. Cultures were subsequently diluted to an optical density of 0.05 at OD_600_in 70:30 medium. Approximately 24 h after the start of stationary phase (T24), 1–2 ml samples were withdrawn from the cultures. The samples were pelleted at a maximum of 21,130 × *g* for 30 s and were subsequently resuspended in 10 µl of double-distilled water. A 2 µl aliquot of each concentrated culture was placed on a thin layer slide, and the *C. difficile* cells were Gramstained and imaged with a Nikon Eclipse Ci-L microscope at 100× magnification. A minimum of 500 cells from each strain were examined to calculate the percentage of spores.

To assess ethanol-resistant spore formation, 500 µl of samples from the sporulation medium were removed from the anaerobic chamber and mixed 1:1 with 95% ethanol for 15 min to kill vegetative cells. The samples were then returned to the anaerobic chamber, 100 µl of the ethanol-treated cultures were mixed with 100 µl of 10% taurocholate, and the mixtures were plated onto BHIS agar to induce *C. difficile* spore germination.The ethanol-resistant CFU/ml was determined after incubation for 24 h and was divided by the total CFU/ml of the nonethanol treated cultures. A minimum of three biological replicates were performed for each strain.

### Germination efficiency of *C. difficile* spores

#### Spore preparation

*C. difficile* strains were cultured in Clospore medium under anaerobic conditions for 4 weeks^53^. For each strain, a 50-ml culture was centrifuged, and the pellet was washed five times to remove the medium, and resuspended in 3 ml of sterile water. The suspension was layered onto the top of 10 ml of 50% (wt/vol) sucrose in water in a 15-ml tube. The gradient was centrifuged at 3200 × *g* for 20 min, after which the spore pellet at the bottom was washed five times to remove the sucrose and was resuspended in water. All spore preparations were >99% pure, free of vegetative cells and debris^54^.

#### Determination of the initiation of spore germination

The initiation of spore germination was monitored aerobically at OD_600_ (the initiation of *C. difficile* spore germination is unaffected by the presence of oxygen)^33^. *C. difficile* spore germination was initiated by suspending spores in the germination solution (10 mM Tris, 150 mM NaCl, 100 mM glycine, and 10 mM taurocholic acid; pH 7.5). Spores were heat-shocked at 65 °C for 30 min and then placed on ice. Next, 5 µl of spores was diluted into 995 µl of buffer with or without germinant and mixed, and the change in optical density at 600 nm (OD_600_) was monitored. The ratio of the OD_600_ at time X (Tx) to the OD_600_ at time zero (T_0_) was plotted against time. The germination rates were determined using the slopes of the linear portions of the germination plots, and the data are reported as the averages from three independent experiments with standard deviations.

#### Determination of Ca-DPA release

Ca-DPA (Calcium dipicolinic acid) release was monitored in real time using a terbium fluorescence assay^55,34^. An opaque 96-well plate was seeded with 125 µl/well of the germination solution supplemented with 800 µM of TbCl_3_. Heat (65 °C)-activated spores were sedimented for 1 min at 14,000 × *g*, and resuspended in an equal volume of water. A 5 µl sample of a spore suspension (with an OD_600_ of 30) was added to each well, and the Ca-DPA release was monitored using a Molecular Devices Cytation™ 5 Imaging Multi-Mode Reader (BioTek, USA) (excitation, 270 nm; emission, 545 nm; cutoff, 420 nm)^55^.

### Motility assay

Motility assays were performed to assess the swimming behavior of the *C. difficile* strains^18,56^. Motility plates were prepared by adding 25 ml of agar medium (37 g/L BHI broth medium, 0.3% (w/v) DifcoBactoagar) into each plate, and the agar was allowed to set overnight. The softagar plates were then air-dried for 10 min in a laminar fume hood with airflow, transferred into the anaerobic cabinet and allowed to reduce for 4 h prior to inoculation with *C. difficile* strains. For each strain, a single colony was inoculated in the middle of a swim plate, which was then incubated at 37 °C for 72 h. Motility was quantitatively determined by measuring the radius of the zone of motility at four different time-points (12, 24, 48, and 72 h). Motility assays were performed in three replicates for each strain, and each assay was repeated independently three times.

### TEM of flagella

Electron microscopy was performed to examine the presence of flagella on the cell surface of the *C. difficile* strains. *C. difficile* cells from mid-exponential cultures were diluted in sterile water and then adsorbed onto carbon formvar copper grids (Agar Scientific Ltd., UK) for 5 min. After air-drying overnight, the grid was quickly washed once with sterile distilled water for 10 s. The air-dried grid was visualized using a JOEL JEM1010 transmission electron microscope operating at 80 kV.

### Biofilm formation of *C. difficile* strains

*C. difficile* strains were cultured overnight in reinforced clostridial medium (RCM, CM0149, OXOID) broth at 37 °C for 24 h in an anaerobic chamber. Next, the culture was diluted 1:100 in RCM broth and vortexed to mix well^36^. The diluted cultures (200 µl) were dispensed into each well of a 96-well microplate and incubated at 37 °C for 5 days in an anaerobic cabinet to allow for biofilm formation. Subsequently, the plates were removed from the anaerobic cabinet, and the planktonic cells and culture supernatant were carefully removed using a Pasteur pipette. An aliquot (200 μl) of 2.5% glutaraldehyde solution was added into each of the drained wells, and the cells were fixed for 30 min. Next, the glutaraldehyde solution was removed, and the fixed biomass was washed with 200 μl of PBS to remove the sedimented planktonic cells. The PBS was discarded, and the wells were stained with 200 μl of a 0.25% (w/v) aqueous CV solution for 10 min. The wells were washed with PBS five times and then were allowed to air dry. The quantity of biofilm formed was determined by adding 200 μl of solvent (1:1 ethanol and acetone solution) to each well to dissolve the dye from adherent cells (biofilm). The absorbance was measured within 5 min of adding the solvent at 570 nm using a Dynex plate reader. The microplate biofilm assay was performed three times for all tested C*. difficile* strains.

### Adhesion assay of *C. difficile* spores

The adherence of the *C. difficile* spores to human gut epithelial cells was assessed as described previously^57^. HCT-8 human gut epithelial cells were obtained from the American Type Culture Collection (ATCC) and were cultured in RPMI 1640 (Gibco) supplemented with 10% (v/v) FBS, 10 mM L-glutamine, and 100 μg/mL penicillin/streptomycin. After incubating for 5 days, the HCT-8 cells (5 × 10^5^ cells per ml in a 6 wells plate) were inoculated with 100 µl of 5 × 10^7^ spores/ml in sterile water, and the plate was incubated for 100 min at 37 °C in an anaerobic chamber. After incubation, the cell-spore mixture was washed three times with 1 × PBS via centrifugation at 800 × *g* for 1 min to remove any unattached spores. The supernatants after centrifugation from each wash step were collected to enumerate any spores that did not adhere to the cells. The spores in the supernatant were enumerated on prereduced BHI agar supplemented with 10% taurocholic acid (w/v). The controls included PBS incubated with spores and RPMI incubated with spores, and the adhesion assays were performed in triplicate. The percentage of spore adherence was calculated using the following formula: (initial CFU/ml – eluted CFU/ml)/initial CFU/ml.

### Determination of TcdA/TcdB production

Free toxin levels were determined at four different time points (12, 24, 48, and 72 h postinoculation). Single colonies of the strains were cultured in BHIS medium for 18–24 h (to stationary phase), and then were subcultured into fresh TY medium (3% (w/v) tryptose, 2% (w/v) yeast extract, 0.1% (w/v) thioglycollate, pH 7.4), adjusted to the same OD_600_ value (0.05)^58^. After incubation, the postculture broth of each time point was placed in a 50-ml centrifuge tube, and centrifuged at 12,000 rpm for 10 min at 4 °C. The supernatants were filtered through a membrane filter with a 0.45-μm pore size, and the toxin concentrations in the supernatants were measured by ELISA. Briefly, 96-well Costar microplates were coated with 100 µl of an anti-TcdA antibody (1 µg/ml) or an anti-TcdB antibody (1 µg/ml) overnight in phosphate-buffered saline (PBS) at 4 °C. The following day, each well was blocked with 300 µl of blocking buffer (PBS + 5% dry milk) at RT for 2 h. Next, standards and samples were added to each well (100 µl) in duplicate, and the samples were incubated for 90 min at 25 °C. After another set of washes, HRP-chicken anti-*C.difficile* TcdA/TcdB (1:5000 dilution in PBS, Gallus Immunotech, Shirley, MA) was added to wells for 30 min at RT. After three washes, a TMB Microwell peroxidase substrate for 20 min at RT in the dark. The reaction was stopped with 2 N of H_2_SO_4_, and the absorbance was measured at 450 nm using a plate reader.

The total toxin concentration at four different time points was also assessed using a *C. DIFFICILE TOX A/B II*™ assay (TechLab, Inc., Blacksburg, VA) according to the manufacturer’s instructions. Positive results for the *C. difficile* TOX A/B II assay are indicated by OD_450/620_ values of ≥0.080, and negative results are indicated by OD_450/620_ values of <0.080.

### Evaluation of the virulence of *C. difficile* strains in mice

Six-week-old C57BL/6 female mice were purchased from Charles River Laboratories, MA. All animal experiments were approved by the Institutional Committee for Animal Care and Use at the University of South Florida. Forty mice were divided into four groups (*n* = 10) to be challenged with spores of strains CD630, R20291, UK6, and LC693. Mice were provided drinking water containing a mixture of six antibiotics, including kanamycin (40 mg/kg), gentamycin (3.5 mg/kg), colistin (4.2 mg/kg), metronidazole (21.5 mg/kg), and vancomycin (4.5 mg/kg) for 5 days. Subsequently, mice were provided autoclaved water for 2 days, followed by a single dose of clindamycin (10 mg/kg) via intraperitoneal injection before being challenged with 10^6^*C. difficile* spores/mouse via oral gavage as described previously^59^. After infection, the mice were monitored daily for a week for survival, weight changes, diarrhea, and other symptoms of disease. Diarrhea was defined as wet tails with loose or watery feces. The number of recorded dead mice included the mice that died after infection and those that were euthanized if weight loss was greater than 20%.

### Statistical analysis

Animal survival was analyzed by Kaplan–Meier survival analysis with a log-rank test of significance. When comparing results for two groups, Student’s unpaired *t*-test was used to assess significance. When comparing more than two groups, one-way analysis of variance (ANOVA) with post hoc analysis by Bonferroni tests was used. The results are expressed as the means ± standard errors of the means. Differences were considered significant if *p* < 0.05 (*). All statistical analyses were performed using GraphPad Prism.
